# Anti-inflammatory *Streptococcus thermophilus* CNRZ160 limits sarcopenia induced by low-grade inflammation in older adult rats

**DOI:** 10.3389/fnut.2022.986542

**Published:** 2022-09-29

**Authors:** Isabelle Savary-Auzeloux, Marianne Jarzaguet, Carole Migné, Jean-Louis Kemeny, Lorraine Novais-Gameiro, Marcela de Azevedo, Véronique Mathé, François Mariotti, Philippe Langella, Jean-Marc Chatel, Dominique Dardevet

**Affiliations:** ^1^Unité Nutrition Humaine, UMR1019, Université Clermont Auvergne, INRAE, Clermont-Ferrand, France; ^2^MetaboHUB Clermont, Plateforme d'Exploration du Métabolisme, Unité de Nutrition Humaine (UNH), Institut National de Recherche pour l'Agriculture, l'Alimentation et l'Environnement (INRAE), Université Clermont Auvergne, Clermont-Ferrand, France; ^3^Centre Imagerie cellulaire et Santé – CICS - Université Clermont Auvergne, Clermont-Ferrand, France; ^4^Université Paris Saclay, AgroParisTech, UMR1319 MICALIS, INRAE, Jouy en Josas, France; ^5^Université Paris-Saclay, AgroParisTech, UMR PNCA, INRAE, Paris, France

**Keywords:** aging, gut, *Streptococcus thermophilus*, muscle, inflammation, DSS

## Abstract

**Background and aims:**

Aging is characterized, at the systemic level, by the development of low-grade inflammation, which has been identified as determining sarcopenia by blunting postprandial muscle anabolism. The causes of this “inflammageing” is still not clearly defined. An increased intestinal permeability, a microbiota dysbiosis and subsequent generation of intestinal then generalized inflammation have been hypothesized. The objective of this study was to test *in vivo* during aging if (1) a chronic low-grade intestinal inflammation can lead to anabolic resistance and muscle loss and (2) if a bacterial strain presenting anti-inflammatory properties could prevent these adverse effects.

**Methods:**

Young adult (6 m) and elderly rats (18 m) received Dextran Sodium Sulfate (DSS) for 28 days to generate low-grade intestinal inflammation, and received (PB1 or PB2 groups) or not (DSS group) one of the two S. Thermophilus strains (5 × 10^9^ CFU/day) previously shown to present an anti-inflammatory potential *in vitro*. They were compared to pair fed control (PF). Muscle and colon weights and protein synthesis (using ^13^C Valine) were measured at slaughter. Muscle proteolysis, gut permeability and inflammatory markers were assessed only in old animals by RT-PCR or proteins quantifications (ELISA).

**Results:**

In both adult and old rats, DSS reduced absolute protein synthesis (ASR) in gastrocnemius muscle [−12.4% (PB1) and −9.5% (PB2) vs. PF, *P* < 0.05] and increased ASR in colon (+86% and +30.5%, respectively vs. PF, *P* < 0.05). PB1 (CNRZ160 strain) but not PB2 resulted in a higher muscle ASR as compared to DSS in adults (+18%, *P* < 0.05), a trend also observed for PB1 in old animals (+12%, *P* = 0.10). This was associated with a blunted increase in colon ASR. In old rats, PB1 also significantly decreased expression of markers of autophagy and ubiquitin-proteasome pathways vs. DSS groups and improved gut permeability (assessed by *Occludin, Zonula Occludens 1 and Claudin 1* expression, *P* < 0.05) and alleviated systemic inflammation (A2M: −48% vs. DSS, *P* < 0.05).

**Conclusion:**

The loss of muscle anabolism associated with low-grade intestinal inflammation can be prevented by supplementation with anti-inflammatory CNRZ160 strain. We propose that the moderated gut inflammation by CNRZ160 may result in curtailed amino acids (AA) utilization by the gut, and subsequent restored AA systemic availability to support muscle protein accretion. Therefore, CNRZ160 could be considered as an efficient probiotic to modulate muscle mass loss and limit sarcopenia during aging.

## Introduction

While life expectancy continues to increase, life expectancy without limited functional capacities has stagnated in most European countries and generates a state of frailty in the older adult population. Frailty is a complex clinical state characterized by the loss of physiological homeostasis and capability to handle modifications of its environment (1, 2). One of the main determinants of frailty is the development of sarcopenia that can appear well before the first clinical signs of mobility loss and dependency. Sarcopenia can represent up to nearly 20% of the general population ≥60 years of age in the world (3). The causes underlying sarcopenia development are multifactorial, including, in among others, decreased physical activity, lower appetite and malnutrition, an impairment/decline of endocrine factors and neuromuscular control, as well as the development of skeletal muscle resistance to the post prandial anabolic effects of nutrients like amino acids (AA) and hormones like insulin (4).

In the elderly population, the presence of a low-grade inflammation, so called “inflammageing”, has been considered to be one of the major determinants of health status in old individuals (5) and suspected of being a favorable environment for the development of sarcopenia (6). Of note, an increased inflammatory status in the elderly population, and particularly higher levels of Interleukin 6 (IL-6) and Tumor Necrosis Factor Alpha (TNF-α) as well as Interleukin 1 (IL-1) and C-reactive protein (CRP) are considered as markers of frailty or predictors of mortality (7). Even if the increase is moderate, higher levels of cytokines and CRP increase the risk of muscle strength loss, of lower aerobic fitness (8), correlated with lower muscle mass in older individuals (9).

The concern with the setting of this low-grade inflammation is that even though it arises gradually with age and remains clinically silent, it can have a significant impact on muscle protein metabolism which controls muscle mass and function. Indeed, we have shown previously in old rats that a slight increased IL6 level as well as an elevation of plasma fibrinogen and alpha-2 macroglobulin (A2M; two positive acute phase proteins) were already associated with a blunted anabolic effect of meal intake on muscle protein synthesis. It has been shown that when it was chronically prevented with non-steroidal anti-inflammatory drugs, muscle protein synthesis response to food intake was maintained in older adult rats with an increased muscle mass status (10). In different acute inflammation situations (11), cytokines have been shown to be also involved in the increase of muscle proteolysis, particularly the ubiquitin proteasome system (UPS) and autophagy and consequently also involved in the acceleration of muscle mass loss in old population (6). All together, these results suggest that anti-inflammatory strategies could participate in the protection of muscle mass and function during aging.

The causes of the development of this systemic low-grade inflammation in the elderly population are still not clearly known. Among the potential causes, is now well established that gut microbiota composition shifts with aging, with both a decreased diversity and an increased ratio of “pathogenic” vs. “beneficial” microbes (12). In this context, there are reports of associations between the inflammatory status of the elderly population, the microbiota composition (13) and the level of dependence of older adults (14). A combination of gut morphologic alteration of the gut (“gut leaking”) along with an altered microbiota composition has been suspected to be one of the causes of inflammageing development (15), including some direct/indirect effects on peripheral tissues, including muscle metabolism (16). However, the causal role of microbiota dysbiosis in low-grade inflammation development at the peripheral levels in elderly remains to be strengthened. However, it could be strongly suspected by the study of Fransen et al. (17) who showed that microbiota transfer from elderly mice to axenic young mice stimulated immune pathways in the gut with bacterial components found in systemic circulation of the recipient mice capable to generate low-grade inflammation (NFκB pathway). Hence, we can speculate the existence of a gut-microbiota-muscle axis that could be also one of the determinant responsible for sarcopenia development during aging (18, 19).

So far, strategies have been proposed including adapted physical activity programs and/or nutritional intervention but have been shown to be non-optimal over time when prescribed alone or even in combination (20). Consequently, strategies capable to down-regulate gut low-grade inflammation could be of relevance in the prevention of sarcopenia in older adults. Hence, the aim of the present work was to determine, in an aged rodent model, if a chronic gut low-grade inflammation could exacerbate sarcopenia and what could be the mechanisms involved in the initiation of this gut-muscle axis. We also aimed at deciphering if ingestion of *Streptococcus thermophilus*, previously selected *in vitro* for their anti-inflammatory properties (21), could limit muscle mass loss in case of slight gut inflammation.

## Materials and methods

### Animal's experiments

The studies were approved by the Animal Care and Use Committee of Auvergne (CEMEA Auvergne; Permit Number: C2EA-02) and the Ministère de l'Enseignement Supérieur et de la Recherche (no. 2016112815417443).

### Bacteria preparation

*Streptococcus thermophilus* CNRZ160 or PB5MJ were grown in M17+ 2% lactose overnight at 42°C. Cultures were centrifuged 10 min, 5,000 g and pellet was washed in Phosphate-Buffered Saline (PBS). Resuspended bacteria were centrifuged again 10 min, 5,000 g. Pellet was then resuspended in PBS + 20% glycerol (5 × 10^9^ CFU/ml final concentration).

### DSS-induced low grade inflammation. Doses consideration

Experimental colitis caused by dextran sodium sulfate (DSS) in rodents is a recognized model of human intestinal inflammatory disease. This colitis occurs within the first days of treatment with DSS in the drinking water with the following main clinical manifestations: diarrhea, bloody stools, weight loss, bleeding and anemia. The type of DSS chains and the diet of rats is known to influence the severity of symptoms. Thus, we tested, using our regular rat chow (SAFE A04, SAFE SAS, France), a DSS batch used in all the experiments described below (MPbio; 160110; M.W. = 36,000–50,000) at different concentrations (from 2 to 4%) in order to obtain sub clinical colitis symptoms (weight loss but without acute diarrhea or bleeding). We performed this study on fifteen old rats and determined the adequate dose of DSS to generate inflammation at a low-grade level (data not shown, see results and discussion sections for details).

### Selection of a probiotic strain capable to counteract DSS-induced muscle mass loss: A study in adult rats

This study aimed at testing whether low-grade intestinal inflammation could be accompanied by significant muscle loss and, if this first assumption was proven true, whether a strategy based on anti-inflammatory probiotics could counteract muscle wasting. We tested two strains of *S. thermophilus* preliminary characterized for their anti-inflammatory properties based on the modulation of IL-10 and IL-12 secretion by mononuclear cells *in vitro* (21).

Wistar adult male rats (6 month-old, *n* = 48; Janvier, Le Genest Saint Isle, France) were housed individually and kept in a controlled environment (temperature maintained at 22°C; 12:12 light: dark cycle). After an adaptation period fed the regular chow (SAFE A04, SAFE SAS), animals were assigned to one of the 4 following groups [*n* = 12 or 13 per group; PF (pair fed controls), DSS (DSS treated), PB1 (DSS + probiotic #1) and PB2 (DSS + probiotic #2)] and homogenized for body weights and lean masses.

According to our preliminary tests, and within the living conditions described above, chronic low-grade intestinal inflammation was then induced by 4% (w/v) DSS in the drinking water supplied in three groups of rats (DSS, PB1 and PB2 groups) for 4 weeks. PB1 and PB2 groups were given orally 5 × 10^9^ CFU of one of the bacterial strains *S. thermophilus* CNRZ160 (PB1) or PB5MJ (PB2) per day. As previous experiments (22) had shown a reduction of food intake in DSS treated animals, the control group was pair-fed (PF) to the DSS treated groups (DSS, PB1 and PB2) to avoid any confounding effect associated with the quantity of food ingested.

Food and drinking intakes were daily monitored in order to adjust the pair feeding and the dose of DSS. Animals were weighted daily and body composition assessed longitudinally using Magnetic Resonance Imaging (MRI; Echo MRI International) before treatment (for groups assignment) and at the end of the experiment (i.e 2 days before sacrifice). Along the experiment, impact of DSS on gut health was assessed *via* the calculation of the disease activity index (DAI) based on body weight, gross rectal bleeding and stool consistency.

At the end of the experiment (28 days), animals were sacrificed in the fed state (i.e., 150 min after the intake of a 6 g meal) using isoflurane gas anesthesia. For protein synthesis measurement, and 40 min before the sacrifice, animals were injected intravenously with L-valine (150 μmol per 100 g body weight) containing 100% of L-[1-^13^C] valine (Euriso-Top, Saint Aubin, France). As anesthetic could alter glucose plasma levels, a blood sample was withdrawn from the tail vein to measure plasma glucose and insulin just before the tracer injection in the waking state. Plasma and insulin levels were then measured 110 min post prandially.

At sacrifice (i.e., 150 min), blood was withdrawn from the abdominal aorta using EDTA as anticoagulant. Blood samples were centrifuged at 2,000 x g for 10 min at 4°C and plasma was frozen in different aliquots for further analysis. Gastrocnemius muscle was rapidly removed, weighed, freeze-clamped into liquid nitrogen and stored at −80°C for further analysis. Colon was removed and the colonic damage was scored on the 0–10 Wallace scale. All the colons of the rats given DSS obtained a score of 0 (No damage) or 1 (Hyperemia – No ulcers) which confirmed that the induced inflammation remained low and subtle even after a month of daily treatment. A 10 mm section of the proximal part of the colon was used for histopathological examination. The rest of the colon tissue was weighed, freeze-clamped into liquid nitrogen and stored at −80°C for further analysis.

### Impact of CRNZ160 to limit muscle mass loss following low-grade gut inflammation: A study in old rats

Twenty months (*n* = 45; old) Wistar male rats (Janvier, Le Genest Saint Isle, France) were housed individually and kept in a controlled environment (temperature maintained at 22°C; 12:12 light: dark cycle). After an adaptation period during fed the regular chow, animals were divided into three groups [*n* = 16 per group: PF (control pair fed), DSS (DSS treated), PB1 (DSS + probiotic CNRZ160)] and homogenized for body weight and lean mass as described above for adult rats. In the same manner as in the study carried out with adult animals, low grade gut inflammation was induced by adding 4% (w/v) of the same batch of DSS to the drinking water for four weeks (DSS and PB1 group). PB1 group received daily 5 × 10^9^ CFU of the bacterial strains *S. thermophilus* CNRZ160. Control of food and water intake, weight and body composition were carried out as previously described in adult animals.

All samplings and injections (^13^C valine for protein synthesis assessment) were carried out as previously described in adult animals. However, additional measurements were performed in old animals. In particular, thorough analyses have been done on plasma and tissue samples for a better evaluation of inflammatory and metabolic status at local and systemic.

### Plasma glucose insulin and inflammatory markers

Plasma glucose level was measured enzymatically using a commercial kit (Horiba, France). Plasma insulin level was assessed using a rat ELISA kit (Mercodia, Sweden). Glucose homeostasis was further estimated using the homeostasis model assessment-estimated insulin resistance (HOMA-IR) using the HOMA2-IR model to calculate an insulin résistance index in our fed state. Plasma concentrations of A2M, Lipopolysaccharide (LPS)-binding protein (LBP), soluble Cluster of Differentiation 14 (sCD14) and alpha-1-acid Glycoprotein (AGP) were measured using commercially available ELISA kits (Clinisciences, Nanterre, France) performed according to the manufacturer's instructions. Plasma concentrations of the cytokines Interleukine 1 beta (IL1β), IL6, IL10, Interleukine 12 p70 (IL12) and Interleukine 17 (IL17) were determined simultaneously by multiplex immunoassay on a Bio-Plex 200 (BioRad, Hercules, CA, USA), using the Bio-Plex Pro™ Rat Cytokine Th1/Th2 Assay (BioRad).

### Gut inflammatory status

A 10 mm section of the proximal part of the colon was cut, collected and fixed in PBS containing 10% paraformaldehyde in a 1.5-ml tube for the histological analysis. Samples were dehydrated and embedded in Paraffin. Paraffin-embeded sections (5 μm) were then stained with haematoxylin and eosin (H&E) using appropriate procedures and examined under a light microcope. A blind-scoring of the H&E-stained colonic tissue sections using a method applied in previous publications (23) as follows: (i) inflammation (0, none; 1, slight; 2, moderate; 3, severe; and 4, accumulation of inflammatory cells in the gut lumen); (ii) extent (0, none; 1, mucosa only; 2, mucosa and submucosa; 3, limited transmural involvement; and 4, transmural); and (iii) percent involvement (0, none; 1, 1%−25%; 2, 26%−50%; 3, 51%−75%; and 4, 76%−100%). The overall histological score was the sum of the three variables, and the maximum score was 12.

Myeloperoxidase (MPO) activity in the colon was assessed by a colorimetric assay and used as an index of inflammation. Briefly, 50 mg of colon were homogenized in 1 ml of hexadecyltrimethylammonium bromide (HTAB) buffer [5 g of HTAB in 1 L of Potassium Phosphate buffer (50 mM, pH = 6.0)] with a tissue lyser (MM400, Retsch GmbH, Germany) for 6 min at 30 Hz at 4°C. The solution was centrifuged for 6 min (13,000 × g, 4°C), the supernatant was collected and 7 μl were added in triplicate into a 96-wells plate. Fifty microliter of diluted H_2_O_2_ (4 μl of 30% H_2_O_2_ diluted in 96 μl of dH_2_O) to a o-dianisidine mixture (16.7 mg *o*-dianisidine dihydrochloride, 90 ml of dH_2_O, 10 ml of potassium phosphate buffer). Two hundred microliter of this o-dianisidine mixture containing H_2_O_2_ were added to each of the wells and absorbance at 450 nm was measured using a spectrometer, three readings were taken at 30 s intervals. MPO activity is measured in units (U) of MPO/mg tissue.

### Protein synthesis in tissues

A 200 mg aliquot of muscle or colon tissues were homogenized in 2 ml of 10% trichloroacetic acid (TCA). Homogenates were centrifuged (5,000 × g, 15 min, 4°C). TCA insoluble materials were washed four times in four volumes of cold 10% TCA. Resultant pellets were resuspended in 0.3 N NaOH and incubated at 37°C for 1 h. Protein concentration was determined using biccinchoninic acid assay (BCA, Pierce). Proteins were hydrolyzed in 6N HCl at 110°C for 48 h. HCl was removed by evaporation and amino acids purified by cation exchange chromatography (AG 50 X 8, 100–200 mesh, H+ form, Biorad, Richmond, CA, USA). Enrichment of [1–^13^C] valine into tissue proteins was measured as its *N*-acetyl-propyl derivatives by gas chromatography–combustion-isotope ratio mass spectrometry (GC-C-IRMS).

The plasma ^13^C enrichment of valine was measured by gas chromatography—mass spectrometry (GC-MS, model HP5975C/7890A, Agilent, Santa Clara, USA) using tertiary-buthyldimethylsilyl derivatives. Briefly, 500 μl of plasma were homogenized in 8 volumes of ice-cold 10% TCA and then centrifuged at 5,000 × g for 15 min at 4°C. The supernatants, which contain free amino acids, were purified by cation exchange chromatography (AG 50 X 8, 100–200 mesh, H^+^ form, Biorad, Richmond, CA, USA) in minidisposal columns. Valine and other amino acids were eluted with 4M NH_4_OH. After evaporation of NH_4_OH under vacuum, free amino acids were resuspended in 0.01 M HCl for subsequent derivatization.

The absolute synthesis rate (ASR: total muscle proteins synthetized) was calculated from the product of the protein fractional synthesis rate (FSR) and the protein content of the tissue and expressed in mg/day. FSR (in %/day) was calculated from the formula: FSR = Sb × 100/Sa × *t*, were Sb is the enrichment at time *t* (minus natural basal enrichment of protein) of the protein-bound valine, t is the incorporation time in day, and Sa is the mean enrichment of plasma valine between the time of injection and t. The mean Sa enrichment was the Sa (*t*1/2) value calculated from the linear regression obtained in tissue between the time of injection and time *t*.

### Genes expression in tissues

Quantitative RT-PCR were performed to measure the expression of cytokines in gut, proteolysis actors in gastrocnemius muscle and colon permeability markers. A 100 mg-aliquot of gut or muscle tissues was homogenized in 1 ml of RNAzol (Sigma-Aldrich, France). Homogenate was centrifuged (10,000 × g, 5 min, 4°C). The supernatant was collected and mixed 15 s with 200 μl of chloroform (Sigma-Aldrich). After a 3 min incubation at room temperature, the homogenate was centrifugated (12,000 × g, 10 min, 4°C) and the aqueous phase was collected and mixed with 600 μl of 70% ethanol. RNA were then purified with a RNeasy kit (Qiagen). A reverse transcription was performed with the High-Capacity cDNA reverse transcription kit (Applied Biosystem). qRT-PCR was performed using the SYBR Power PCR Master Mix (Applied Biosystems) according to the manufacturer's instructions using a cFX96 thermocycler (BioRad). Calculations were made using the comparative ΔΔCt method with *YWHAZ, HPRT1* and *36B4 reporter genes* (see Table 1).

**Table 1:** Primers sequences used for qRT-PCR in the colon and the muscle.

**Gene**	**Forward primer**	**Reverse primer**
*IL10*	5′-GTTGCCAAGCCTTGTCAGAA-3′	5′-GGGAGAAATCGATGACAGCG-3′
*IL12-a*	5′-CCCAAAACCTGCTGAAGACC-3′	5′-AGGCACAGGGTCATCATCAA-3′
*IFNG*	5′-CGTCTTGGTTTTGCAGCTCT-3′	5′-TCGTGTTACCGTCCTTTTGC-3′
*TNFA*	5′-CGTCGTAGCAAACCACCAAG-3′	5′-GAGGCTGACTTTCTCCTGGT-3′
*IL1B*	5′-TTTGAAGAAGAGCCCGTCCT-3′	5′-TGTCGTTGCTTGTCTCTCCT-3′
*IL6*	5′-CCACTGCCTTCCCTACTTCA-3′	5′-CCATTGCACAACTCTTTTCTCA-3′
*IL17*	5′-TTGCTGCTACTGAACCTGGA-3′	5′-TCCTCATTGCGGCTCAGAG-3′
*IL18*	5′-CTCTTGGCCCAGGAACAATG-3′	5′-CAGGCGGGTTTCTTTTGTCA-3′
*OCLN*	5′-CGCCTCTGGTACCTGAAGTA-3′	5′-ACCTGTCGTGTAGTCGGTTT-3′
*ZO-1*	5′-TCCTCCTCGACCTCCCTAAA-3′	5′-ACTGCTCGGCTCTGTTCTTA-3′
*CLDN1*	5′-AAAGACTACGTGTGACAGCG-3′	5′-AGCAGCAGTTCAAAGGCAAA-3′
*CLDN15*	5′-CTGTGCCACCGACTCCCTGG-3′	5′-CTAGGCATGGTGGGGCTC-3′
*CLDN2*	5′-GCCTCACAGAGAACCATCCT-3′	5′-CACTGCCAAGGTGTTCTGG-3′
*CLDN3*	5′-ATGGGAACTGGGTTGTACGT-3′	5′-AGTCCTTACGGTCATAGGCG-3′
*CLDN4*	5′-CATCAGCATCATCGTGGGTG-3′	5′-TGTGATCATGACCTTGGCCT-3′
*MUC2*	5′-GAGTTGTATGTGCTCGCCTG-3′	5′-TTTCTTGGGGCAGAGAGAGG-3′
*ATG16L*	5′-AGGAAGAGGCACGCGACTTG-3′	5′-GCCCTCTCTCTACGCTCGTT-3′
*CTSL*	5′-GGTGGGGCCTATTTCTGTTG-3′	5′-TCGAGGTCCTTGCTGCTACA-3′
*LC3b*	5′-CCGGAGCTTCGAACAAAGAG-3′	5′-CAGCTGCTTCTCACCCTTGT-3′
*FbxO30*	5′-TGGCACAAGTCAGAATGCTC-3′	5′-CAGCTTCCACACAGTCTCCA-3′
*MAFbx*	5′-ATGCACACTGGTGCAGAGAG-3′	5′-TGTAAGCACACAGGCAGGTC-3′
*MuRF1*	5′-GTCCATGTCTGGAGGTCGTT-3′	5′-GTCTTCGTGTTCCTTGCACA-3′
*HPRT1*	5′-GCAGACTTTGCTTTCCTTGG-3′	5′-TCCACTTTCGCTGATGACAC-3′
36B4	5′-TTCCTAGAGGGTGTCCGCAAT-3′	5′-GCAACAGTCGGGTAGCCAAT-3′
*YWHAZ*	5′-TTGAGCAGAAGACGGAAGGT-3′	5′-CCTCAGCCAAGTAGCGGTAG-3′

*IL10, Interleukin 10; IL12-a, Interleukin 12A, IFNG, Interferon Gamma; TNFA, Tumor Necrosis Factor Alpha; IL1B, Interleukin 1 Beta; IL6, Interleukin 6; IL17, Interleukin 17;IL18, Interleukin 18; OCLN, Occludin; ZO-1, Zonula Occludens 1; CLDN1, Claudin 1; CLDN15, Claudin 15; CLDN2, Claudin 2; CLDN3, Claudin 3; CLDN4, Claudin 4; MUC2, Mucin 2; ATG16L, Autophagy Related 16; CTSL, Cathepsin L, LC3b, Microtubule-associated protein 1 light chain 3 beta; FbxO30, F-Box Protein 30; MAFbx, Muscle Atrophy F-Box Protein; MuRF1, Muscle-Specific RING Finger Protein 1; HPRT1, Hypoxanthine Phosphoribosyltransferase 1; 36B4, Acidic ribosomal phosphoprotein P0; YWHAZ,14-3-3 protein zeta/delta*.

### Statistical analyses

All data were expressed as means ± SEM. The differences between PF, DSS, PB1 and PB2 groups were analyzed using one-way ANOVA followed by *post hoc* analysis using the Tuckey test (R Studio, Version 1.2.5001, R Studio Inc). When normality of data failed (following a Shapiro Wilk normality test), difference between groups was assessed using a Kruskal-Wallis multiple comparison (*P*-values adjusted with the Bonferroni method) followed by a *post hoc* analysis using Dunn's test (R Studio, Version 1.2.5001, R Studio Inc). Differences were considered significant if *P* < 0.05 and as a tendency (*t*) for 0.05 < *P* < 0.10.

## Results

### Preliminary study on adult rats (6 month old)

The aim of this first study on adult animals was (1) to determine if a slight gut inflammation, by impacting on gut metabolism, could also lead to a significant muscle loss on a long term basis and, if proven true, (2) to test *in vivo* on gut and muscle metabolisms, the beneficial effect of two probiotics from streptococcus genus previously screened *in vitro* for their potential anti-inflammatory properties (21).

#### Food intake and body weight changes

DSS treatment led to a decreased food intake of the animals (34% decrease in average food consumption for all groups vs. *ad libitum* intake measured before the beginning of the experimental period). Consequently, the control group was pair fed (PF) to the DSS treated groups (DSS, PB1 and PB2). The animals from PF, DSS, PB1 and PB2 groups ate, respectively 19.3 ± 0.2, 18.7 ± 0.5, 18.4 ± 0.7 and 18.8 ± 0.3 g of food overall the 27 days of treatment. The food consumption was similar between groups, implying that any difference observed further between groups cannot be attributed to a difference in protein or energy intake.

**Figure 1:**
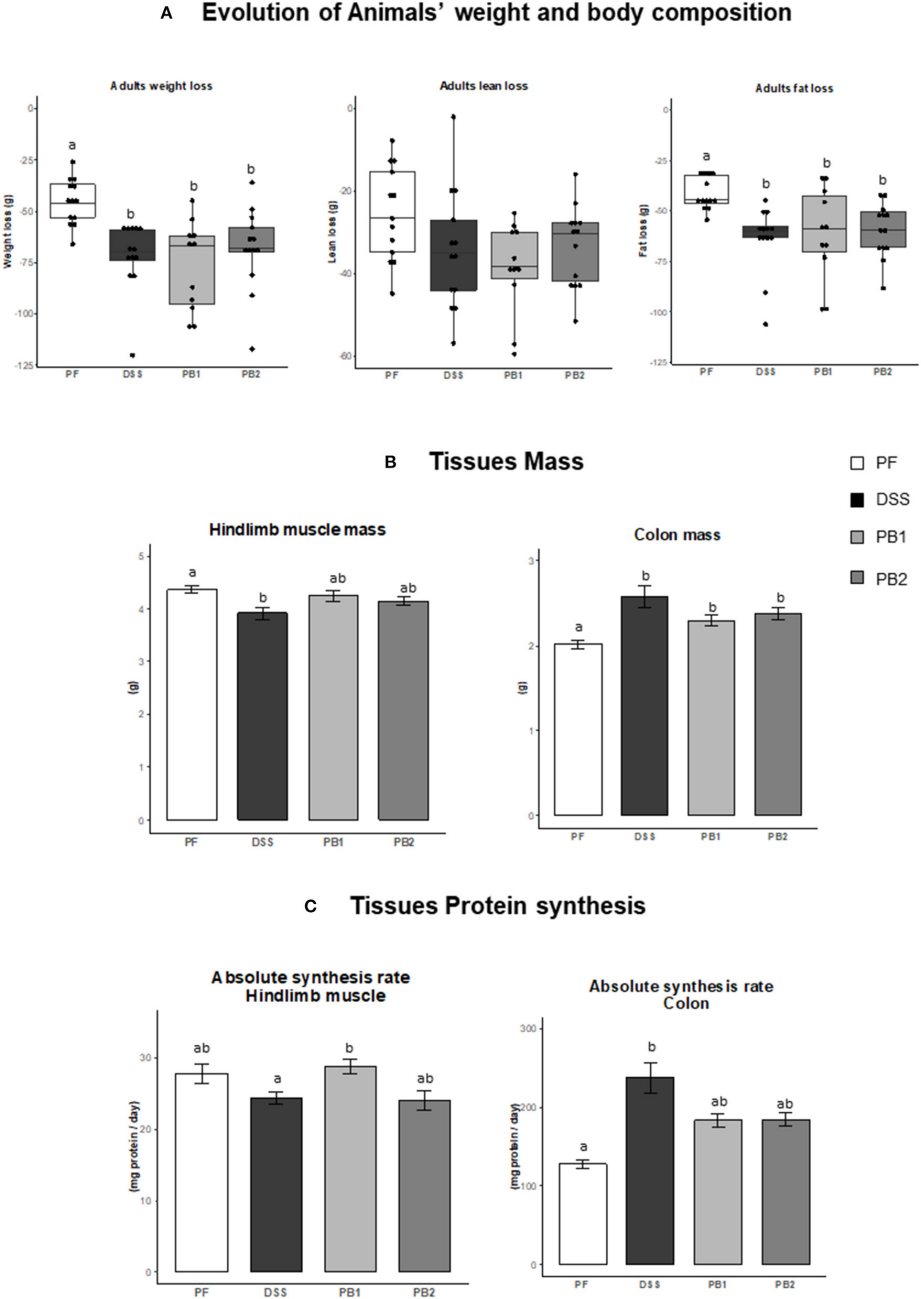
Impact of Dextran sodium sulfate with (PB1 or PB2) or without (DSS) probiotic supplementation on colon and muscle physiology / metabolism in adult rats. **(A)** Evolution of rats weight (g), lean mass (g) and fat mass (g), **(B)** Evolution of hindlimb and colon masses (g), **(C)** Evolution of colon and gastrocnemius absolute synthesis rats (mg protein/ day). Values are means ± SEM different letters indicate significant differences between groups (*P* < 0.05) and were obtained by one way Anova or Wilcoxon rank sum test. Number of samples are 13, 13, 11 and 13 in PF, DSS, PB1 and PB2 groups, respectively for **(A)** and 12, 13, 12, 13 in PF, DSS, PB1 and PB2 groups, respectively for **(B)** and **(C)**.

In the PF group, a decrease of body weight was recorded (i.e., 7.7 ± 0.4% at d27 vs. the mass of the animals at the beginning of the experiment d0). This decreased mass (in %) was significantly exacerbated in DSS treated rats [11.7 ± 1.5, 12.2 ± 0.7 and 11.6 ± 0.6% for DSS, PB1 and PB2, respectively, (*P* < 0.05, Figure 1A)] with no difference between all the DSS groups. The weight loss of the animals was explained by both lean mass and fat mass losses (Figure 1A). Whereas lean mass loss over the experimental period was similar between PF, DSS, PB1 and PB2, fat mass loss was significantly more important in DSS treated animals (DSS, PB1 and PB2 groups) vs. PF (Figure 1A, *P* < 0.05) but not significantly different between the DSS treated groups.

#### Tissue weight and protein synthesis

The hindlimb muscle mass was obtained with the sum of the masses of the 4 main rear leg muscles: Gastrocnemius, Tibialis anterior, Extensor Digitorum Longum and Soleus (Figure 1B). DSS treatment led to a significant muscle atrophy (−10.3% vs. PF; *P* < 0.05). However, when probiotics were given simultaneously with DSS, muscle atrophy was less pronounced and was intermediate between DSS and PF animals. Indeed it was no more different from PF animals (−3.0 and −5.0%, *P* > 0.05 for PB1 and PB2 vs. PF, respectively) and was not different from DSS group (Figure 1B).

On the contrary, colon mass was significantly increased following DSS treatment (+28% for DSS vs. PF, *P* < 0.05; Figure 1B). Probiotics supplementation limited the impact of DSS treatment with a lower but still significant increase in colon mass (+14.4 and +18.4 % for PB1 and PB2, respectively vs. PF, *P* < 0.01; Figure 1B).

ASR measured in gastrocnemius muscle was not decreased significantly in DSS vs. PF groups (*P* = 0.16; Figure 1C). On the contrary, ASR was higher in PB1 than DSS (+18%, *P* < 0.05) suggesting that PB1 was able to improve muscle protein synthesis during the DSS treatment. Interestingly, this was not the case for PB2 that presented a muscle ASR equivalent to the DSS values (24.03 vs. 24.31 mg proteins synthetized/day for PB2 and DSS groups, respectively) and which remained significantly below muscle protein synthesis recorded with PB1 treatment (*P* < 0.05). At the colon level, alteration of protein synthesis is in line with what observed for evolution of its mass. An increased (+86%, DSS vs. PF *P* < 0.05) colon ASR following DSS treatment was not blunted by the two probiotics without any differential effects between the two probiotics studied.

Because our main aim was to evaluate the impact of low-grade gut inflammation on sarcopenia development in elderly, we focused the second study in older adults with the probiotic that showed both potential beneficial effect on muscle mass and muscle protein metabolism in adults. This is why our choice fell on PB1 which, unlike PB2, presented a clear beneficial effect on muscle ASR.

### Study on older adult rats (18 months)

#### Food intake and body weight changes/pathology index

**Figure 2:**
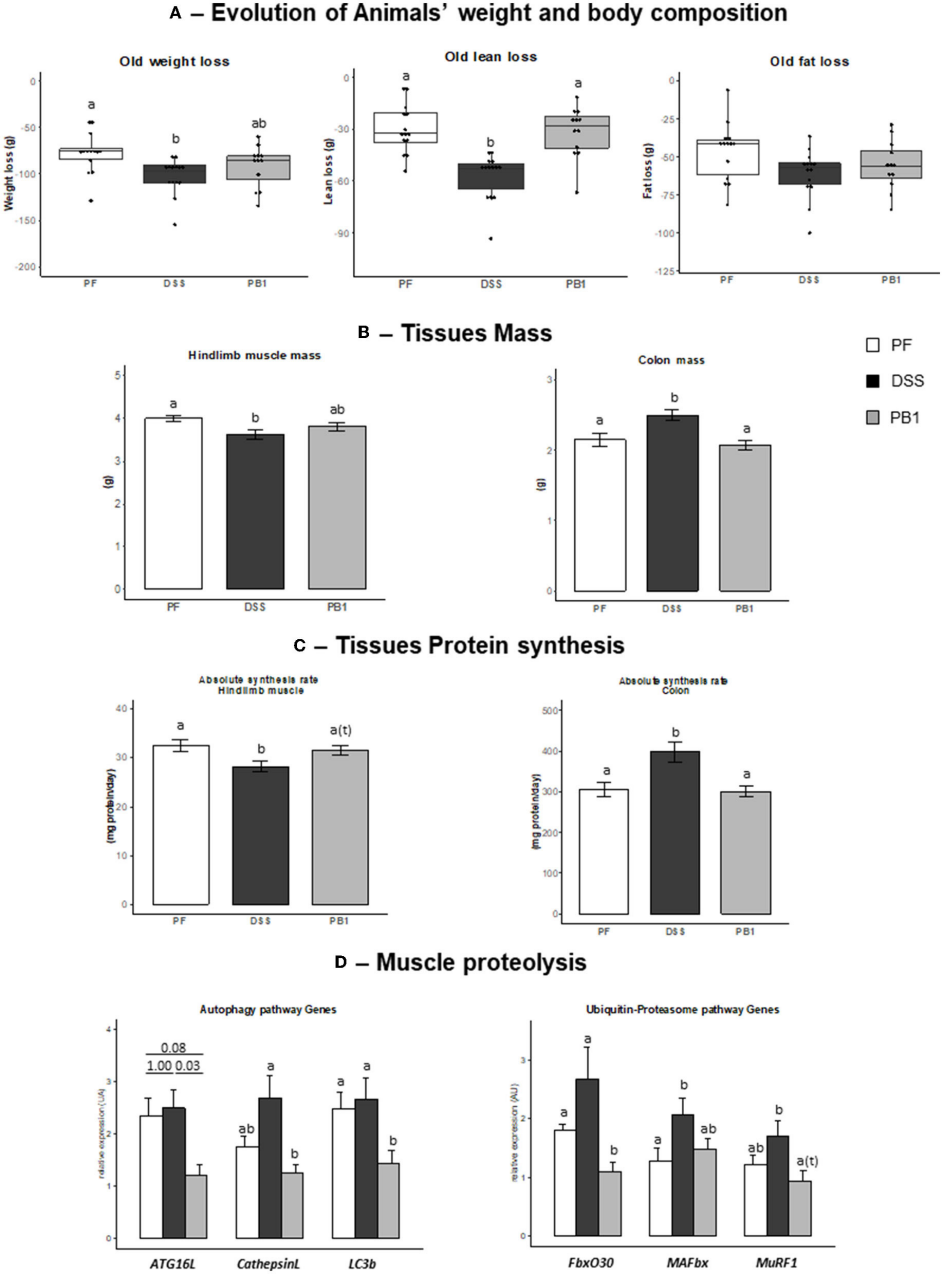
Impact of Dextran sodium sulfate with (PB1) or without probiotic supplementation (DSS) on colon and muscle physiology/protein metabolism in adult older rats. **(A)** Evolution of rats weight (g), lean mass (g) and fat mass (g), **(B)** Evolution of hindlimb and colon masses (g), **(C)** Evolution of colon and gastrocnemius absolute synthesis rats (mg protein/ day), **(D)** Evolution of mRNA levels of muscle proteins involved in autophagy and Ubiquitin-proteasome pathways or regulation of these pathways. Values are means ± SEM different letters indicate significant differences between groups (*P* < 0.05) and were obtained by one way Anova or Wilkoxon rank sum test. Number of samples are 14, 14, 12 in PF, DSS and PB1 groups.

As for adult rats, DSS treatment led to a decreased voluntary food intake (−26%) in all groups of animals, so a pair-feeding was also conducted in older adult rats. During the 27 days of the experimental period, animals ate 18.1 ± 0.2, 17.5 ± 0.5 and 17.8 ± 0.4 g in control PF, DSS and PB1 groups, respectively. Intake was not different between groups, consequently any difference observed in the parameters discussed further cannot be attributed to a difference of food intake. A decreased body weight, lean and fat masses were observed in the PF group (−86 ± 4, −38 ± 3, and −48 ± 3 g, respectively; Figure 2A). However, DSS treatment led to a significant further decreased in body weight and lean masses compared to PF (+27 and +35, respectively, DSS vs. PF, *P* < 0.05; Figure 2A). Probiotics supplementations in DSS treated animals allowed the maintenance of the lean mass at the level of pair fed (PB and PF not significantly different) and limited the body weight loss (PB1 not significantly different from both PF and DSS groups).

**Figure 3:**
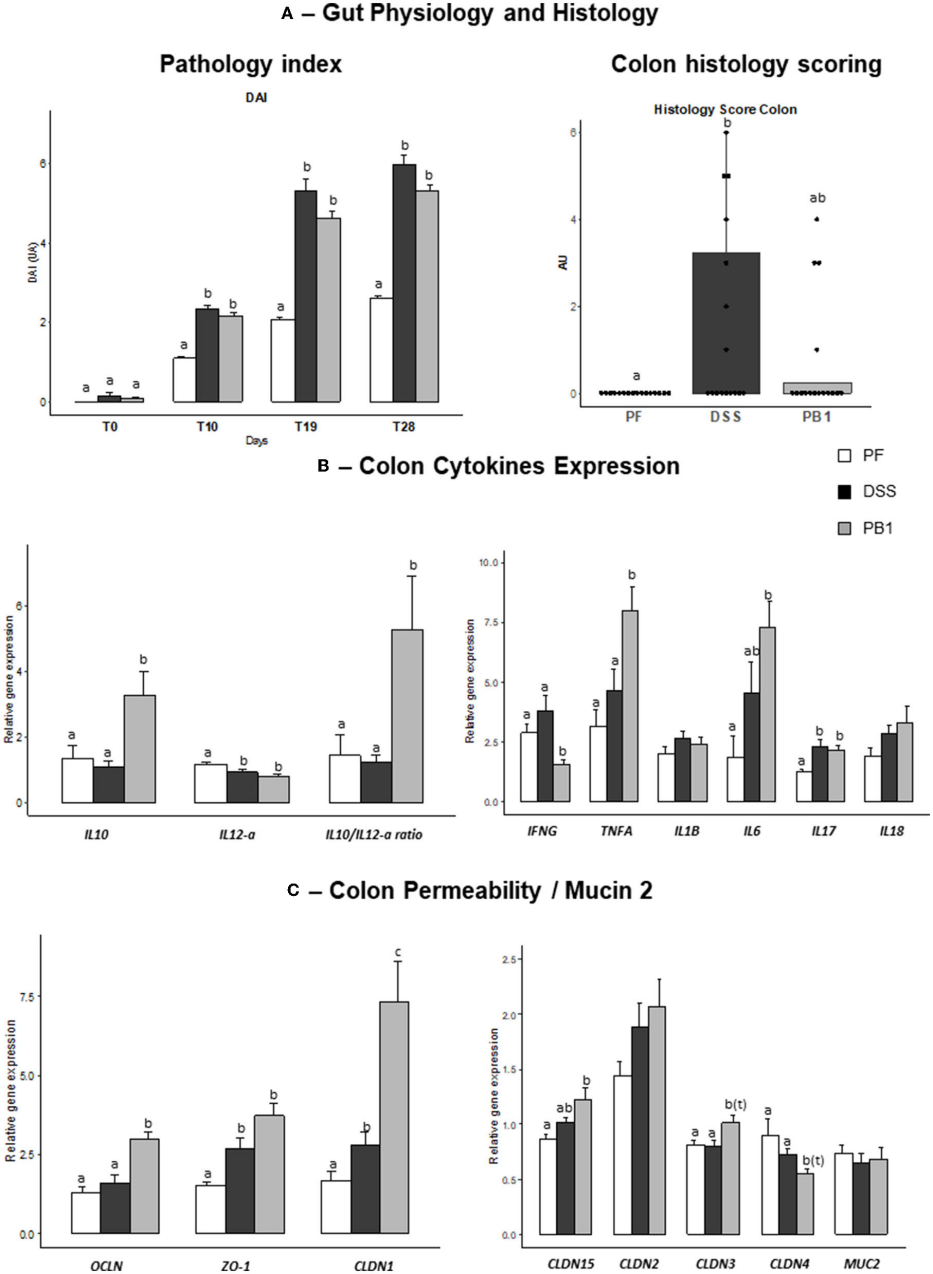
Impact of Dextran sodium sulfate with (PB1) or without probiotic supplementation (DSS) on colon physiology, histology and mRNA levels in adult older rats. **(A)** Evolution of rats pathology index (DAI; AU) and colon histology scoring (AU), **(B)** Evolution of rats colon cytokine mRNA levels (AU), **(C)** Evolution of rats colon permeability and Mucin 2 mRNA levels (AU). Values are means ± SEM different letters indicate significant differences between groups (*P* <0.05) and were obtained by one way Anova or Wilkoxon rank sum test. Number of samples are 16, 16 16 in PF, DSS and PB1 groups for **(A)**, 14, 14, 13 in PF, DSS and PB1 groups for **(B)** and **(C)**. *IL10*, Interleukin 10; *IL12-a*, Interleukin 12A, *IFNG*, Interferon Gamma; *TNFA*, Tumor Necrosis Factor Alpha; *IL1B*, Interleukin 1 Beta; *IL6*, Interleukin 6; *IL17*, Interleukin 17; *IL18*, Interleukin 18; *OCLN*, Occludin; *ZO-1*, Zonula Occludens 1; *CLDN1*, Claudin 1; *CLDN15*, Claudin 15; *CLDN2*, Claudin 2; *CLDN3*, Claudin 3; *CLDN4*, Claudin 4; *MUC2*, Mucin 2.

To evaluate the severity of alteration of gut physiology following DSS treatment, the score of pathology (DAI) was used. A significant increased DAI index was observed in DSS treated animals vs. PF from d10 until the end of the experimental period (Figure 3A). This was also the case for PB1 animals. Of note, the absolute value for DAI found in DSS treated animals (score below 6) is lower than what is generally reported in chronic or acute inflammation generated by DSS in the literature (scores between 8 and 10). Furthermore, because of the pair feeding, the PF group also showed an increased DAI score during the experimental period because of the body weight loss (score around 2, Figure 3A). So, the DAI score recorded in the DSS groups due directly to the treatment is then only around 4. Because we aimed at reproducing a low/moderate grade but not acute inflammation at the gut level, these results suggest that we succeed at the clinical level and it has been comforted by the measurement of the inflammatory markers at the gut and plasma level (see below).

#### Gut and systemic inflammation/Insulin sensitivity parameters

All the colons of the rats given DSS obtained a Wallace score of 0 (No damage) or 1 (Hyperemia – No ulcers) which confirmed that the induced inflammation remained low and subtle even after a month of daily treatment. The occurrence of a mild gut inflammation following DSS treatment was confirmed by the blind-scoring of the H&E stained colon sections. Indeed, scoring level was significantly but only slightly increased (from 0 in PF to 1.5 in DSS) following DSS treatment in our study whereas it reaches the levels of 5 to 25 after 10 days of increasing doses of DSS using the same scoring (23) or multiplied by 6 to 10 vs. control using other scorings methods (24) in other studies. Concerning colonic MPO activity, our data are consistent with results previously obtained by Chassaing et al. (25) following administration of DSS in drinking water (i.e., 5.2 U/mg colon tissue in DSS vs. 1.7 U/mg in control group). MPO activity levels remained similar following PB1 supplementation (5.3 U/mg colon tissue).

**Figure 4:**
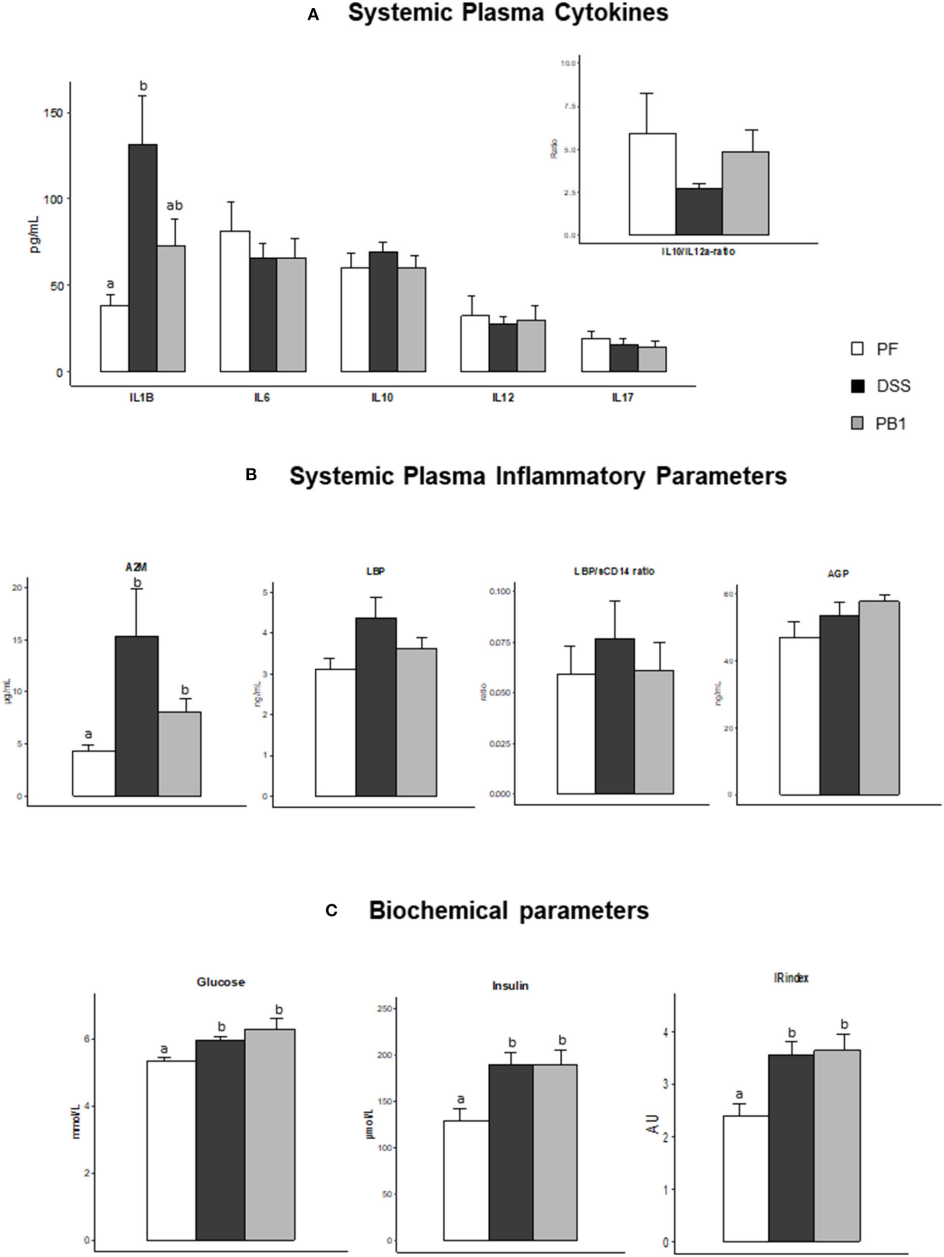
Impact of Dextran sodium sulfate with (PB1) or without probiotic supplementation (DSS) on insulin sensitivity status and systemic inflammation in adult older rats. **(A)** Evolution of rats' plasma cytokines levels: IL1B, IL6, IL10, IL12p70, IL17 and IL10/IL12 ratio, **(B)** Evolution of rats plasma alpha 2 macroglobulin (μg/ml), LBP (ng/ml), LBP/sCD14 ratio (UA), and AGP (ng/ml), **(C)** Evolution of rats glucose (mmol/L), insulin (μmol/L) and Insulin resistance index (AU). Values are means ± SEM different letters indicate significant differences between groups (*P* < 0.05) and were obtained by one way Anova or Wilkoxon rank sum test. Number of samples are 7, 11,11 in PF, DSS and PB1 groups for **(A)**, 14, 14, 13 in PF, DSS and PB1 groups for **(B)** and 14, 14, 12 in PF, DSS and PB1 groups for **(C)**. *A2M*, Alpha 2 Macroglobulin; *LBP*, Lipopolysaccharide Binding Protein; *sCD14*, soluble Cluster of Differentiation 14; AGP, alpha-1-acid Glycoprotein, IL1B, Interleukine 1 beta; IL6, Interleukine 6; IL 10, Interleukine 10; IL12, Interleukine 12 p70; and IL17, Interleukine 17.

At whole body level, DSS treatment was not associated with a modification of the systemic levels of IL6, IL10 IL12a or IL17 (nor the IL10/IL12a ratio) and probiotic supplementation had no effect either with values not significantly different from those of the PF (Figure 4A). However, IL1β showed a significant increase (+243%, DSS vs. PF, *P* < 0.05) which tended to be prevented by the probiotic treatment (Figure 4A). Similarly, A2M was increased after DSS treatment (+257%, *P* < 0.05 vs. PF) and the probiotic limited this increase (only +86%, *P* < 0.05 vs. PF, even if not different from DSS; Figure 4B). No significant effects of the DSS treatment nor the probiotic ingestion was recorded on LBP, LBP/sCD14 ratio and AGP levels (Figure 4B). Metabolically, DSS led to the development of a marked insulin resistance, as shown by the increased glucose, insulin plasma concentrations (and insulin resistance index) in DSS group vs. control (*P* < 0.05). However, the probiotic supplementation did not mitigate insulin resistance (PB1 showing values similar to DSS; Figure 4C).

In the colon, we observed an increased expression of pro-inflammatory *IL17* (+87% DSS vs. PF, *P* < 0.05) but no effect on *IL6, Interferon gamma (IFN*γ*), TNF*α, *IL18* and *IL1*β (Figure 3B). The probiotic ingestion led to an increase in *IL10* gene expression (+199%, PB1 vs. DSS, *P* = 0.005) and in *IL 10/IL12a* genes expression ratio (+327%, PB vs. DSS, *P* = 0.0046), as also shown previously *in vitro* (22), confirming the anti-inflammatory properties of PB1. Levels of expression of *IFN*γ were significantly decreased following PB1 ingestion (−59% vs. DSS, *P* = 0.0006) whereas for *TNF*α and *IL6* (Figure 3B), an increase was recorded (+72%, *P* = 0.008 and + 60%, *P* = 0.048, respectively).

#### Colon tight junction parameters

Dextran sodium sulfate treatment increased the expression of *Zonula Occludens 1 (ZO-1)* and *Claudin 1 (CLDN1*; +79%, *P* = 0.003 and +67%, *P* = 0.03 vs. PF, respectively). No significant effects were found for all the other proteins involved in intestinal permeability regulation [such as *Occludin (OCLN), CLDN 2, 3*, and *4*]. On the contrary, the expression of nearly all these parameters were significantly further increased following PB1 ingestion i.e +127%, +145%, +340%, +42%, +25%, PB1 vs. PF for *OCLN, ZO-1, CLDN1, CLDN15* and *CLDN3*, respectively, *P* < 0.05) suggesting a potential decreased intestinal permeability in PB1 group vs. both PF and DSS groups (Figure 3C). Only *CLDN4* followed an opposite trend following probiotic supplementation (*P* < 0.1 vs. PF and DSS, Figure 3C).

#### Muscle mass, protein synthesis and breakdown

As observed in the adult rats, DSS treatment in older adult rats led to a significant decreased hindlimb muscle mass (−9.5%, *P* < 0.05) vs. PF that can be explained by a significant decreased protein synthesis (ASR −13.4%, *P* < 0.05; Figures 2B,C). Genes expression of molecules involved in autophagy or ubiquitin-proteasome dependent pathways i.e., *Autophagy Related 16 (Atg16L), Cathepsin L, Microtubule-associated protein 1 light chain 3 beta (LC3b), F-Box Protein 30 (FbxO30)* and *Muscle-Specific RING Finger Protein 1 (MuRF1)*, were not significantly modified by the DSS treatment, excepted for *Muscle Atrophy F-Box Protein (MAFbx)* expression (*P* = 0.042, Figure 2D). PB1 ingestion limited DSS induced muscle mass loss (−4.5% vs. PF, not significant) which could be explained by the maintenance of muscle protein synthesis at the level of PF and a significant decrease in the expression of genes involved in autophagy pathway like *ATG16, Cathepsin L, LC3b* that were blunted (−81, −54, −47, −59% in PB1 vs. DSS groups, respectively). This is also the case for *FbxO30* and *Murf1* gene expression (−59 and −45% in PB1 vs. DSS, *P* < 0.05 and *P* = 0.08, respectively; Figure 2D).

#### Colon mass and protein synthesis

As already observed in adult rats' experiment, DSS treatment in older adult rats increased both colon mass and protein synthesis (+16.8% and +30.5% vs. PF, respectively, *P* < 0.05; Figures 2C,D). PB1 treatment reversed the DSS effect and the value for colon mass and protein synthesis were maintained at the level of PF animals, suggesting that the impact of DSS on colon mass and ASR was entirely blunted by the PB1 ingestion.

## Discussion

Sarcopenia development in elderly population have been shown to be linked to the presence of chronic low-grade inflammation (4). However, because the origin of the inflammageing remained hypothetic, the first aim of the present work was to validate the hypothesis that a slight increased inflammation located at the gut/colon level could be one of the causes involved in sarcopenia development during aging. As our data confirmed this preliminary hypothesis in older rodent, our second aim was to prevent the development of this local inflammation and to evaluate if this was sufficient to limit muscle mass loss in aging organisms. Among the possible strategies capable to limit inflammatory processes in the gut, we chose the probiotic approach and selected two strains among candidates screened previously *in vitro* for their anti-inflammatory properties (21). This choice was driven by the recent rich literature suggesting that microbiota dysbiosis observed during aging could be responsible for the development of low-grade inflammation (17). Consequently, anti-inflammatory probiotics could complement already existing nutritional strategies to limit sarcopenia, particularly within the frail population (with low appetite and reduced physical activity). Our data showed that *S. thermophilus* CNRZ160 (26) is capable to limit inflammation and associated muscle loss and thus could be a promising preventive or therapeutic strategy to sustain muscle mass in old population subjected to low-grade gut inflammation.

### Low grade gut inflammation and consequences on muscle protein metabolism

To generate gut-located inflammation, we used DSS diluted in the drinking water. DSS treatment is generally used to mimic intestinal bowel disease (IBD) characterized by an acute but intense gut inflammation, a reduction of colon length, DAI values above 10 and significant increased plasma levels of pro-inflammatory cytokines (TNFα, IL1β, IFNγ) (27). We adapted the dose of DSS to generate a chronic low-grade inflammation with subtle clinical outcomes. The DAI values after DSS remained low, colon length unchanged and minor alterations of colon histologic score or gut cytokine expression and plasma cytokine levels were observed. But the three times increase in plasma level of acute phase protein A2M shows that the treatment did generate a low-grade systemic inflammation. We previously characterized in rats the changes in plasma A2M levels during aging and the present values are in the low range of what it is naturally observed when old adult rodents developed a low-grade systemic inflammation (10).

Among the mechanisms that could be responsible for the loss of muscle mass, two of them could operate. We and others have clearly established that peripheral AA availability is a major determinants of post-prandial muscle protein synthesis stimulation (3, 20). This is a key issue in old population that presents an anabolic resistance due to an impaired response of muscle to post-prandial anabolic signals [dietary AA for the stimulation of protein synthesis or insulin for the inhibition of proteolysis (28)]. Additionally, an increased splanchnic extraction of dietary AA has been demonstrated during aging (29) could limit the peripheral availability of AA (28). Our data suggest that during aging, an increased splanchnic extraction of dietary AA could occur following a gut low-grade inflammation as we showed that colon protein synthesis was dramatically increased, which could represent a significant amount of dietary AA taken up by the colon and therefore not available for the other tissues. This is in accordance with the results obtained in IBD in whom increased distal gut protein synthesis has been shown to compensate the increased losses of amino nitrogen in the gut lumen (30). This is generally accompanied with a blunted growth in young growing IBD patients (30) that could arise from this gut-muscle competition for AA utilization. The second mechanism involves the onset of a systemic low-grade inflammation. We have previously shown in old rats presenting similar increased in plasma A2M levels, an inhibition of post prandial muscle protein synthesis (10) whereas it is normally stimulated in adult rodents (31) or not inflamed old animals (32). In animal models and humans, this blunted muscle protein synthesis was also observed in sepsis that is, however, a much more intense systemic inflammatory situation (22, 33). Muscle mass is also dependent on the rate of proteolysis frequently observed in various pathologies in which systemic inflammation is increased (34) such as burn, cancer, sepsis or IBS model in mice. In all these metabolic situations, ubiquitin proteasome system and/or autophagy/lysosomal proteolysis (35) were up-regulated. The low grade of inflammation induced in our animal model resulted in a trend for a stimulation of *MAFbx* gene expression but not for the other genes tested, suggesting that muscle proteolysis, as inflammation, was, at the most, only slightly stimulated in our study. This is in line with recent data obtained on mice where a 14 days DSS treatment also increased muscle mRNA expression of *MuRF1* and *MAFbx* genes (36).

### An anti-inflammatory bacteria capable to limit muscle mass loss

Because we demonstrated that the low-grade inflammation initiated in the gut had deleterious effects on skeletal muscle in older adult rats, we hypothesized that an oral probiotic treatment could limit its impact on the development of sarcopenia during aging. Two strains from *S. thermophilus* species (CNRZ160 and PB5MJ) have been chosen in the study carried out in adult rats subjected to low-grade gut inflammation. This preliminary *in vivo* study aimed at screening the bacteria that was the most efficient to limit muscle mass loss. Both strains limited the stimulation of colon protein synthesis induced by low grade gut inflammation but only *S. thermophilus* CNRZ160 was able to prevent the decrease of muscle protein synthesis. This confirms the strain-specific impact of probiotics on gut health parameters in chemically induced gut inflammation, some strains from Lactobacillus genera being already proven either beneficial, neutral or detrimental in DSS induced colitis model (37). Because it was proven more efficient to alleviate DSS-induced muscle loss, CNRZ160 strain only was tested in older adult animals.

As observed in adults, CNRZ160 supplementation in older rats limited DSS-induced muscle mass loss and additionally limited whole-body lean mass loss resulting in a better preservation of body composition during the development of low-grade gut inflammation. As for adults, we showed that this could be well explained by a maintenance of muscle protein synthesis during the post prandial phase to the level of PF controls. In parallel, we also demonstrated an inhibition of muscle proteolysis pathways *via* a down regulation of autophagy (*Atg16L, Cathepsin L and LC3b*) and key regulatory elements of Ub-Proteasome pathways (*FbxO30 and MurRF1*) in probiotic treated animals. These combined effects may have concurred to maintain a better anabolic effect during the fed period and then limited muscle mass loss. This suggests that *S. thermophilus* CNRZ160, selected *in vitro* for its anti-inflammatory potential, has been able to handle gut DSS-induced inflammation. We have previously shown that inhibition of the natural low-grade inflammation development in older adult rats (10) was associated with an improvement of post-prandial muscle anabolism, especially on protein synthesis. This was confirmed in our study as CNRZ160 strain allowed a normalization of the acute phase protein A2M secreted by the liver. In contrast, because of the inflammatory condition is a low-grade inflammation, it remains difficult to evidence any effect of probiotic treatment on systemic cytokines as they were not significantly modified beforehand. Still, at the gut level, our probiotic led to an increased expression of anti-inflammatory cytokines genes expression in colon (*IL10, IL10/IL12a* ratio), in accordance with what was evidenced *in vitro* during its selection, along with a decreased expression of *IFN*γ.

Systemic inflammation can arise from an increased permeability of colon and an entry of bacteria in systemic circulation (37). CNRZ160 has probably improved the colon permeability as judged by the increased expression of several tight junction proteins like *OCLN, ZO 1, CLDN 1,3* and *15*. This effect was also reported with other probiotics (essentially some lactobacilli, bifidobacteria, *Akkermansia muciniphila* or probiotics mixture-VSL#3) in models of acute colitis characterized by an acute high grade systemic inflammation (37). Taken all together, we propose that in old adult rodents presenting a low-grade gut inflammation, CNRZ160 strain has ameliorated the colon anatomical integrity by enhancing gut wall permeability and improved the inflammation status at both the colon and the systemic levels.

As discussed above, we have shown that the inflammation, even if it is of low-grade, caused an increase in the mass of the colon associated with a strong increase in the protein synthesis in this organ. The administration of CNRZ160 prevented this increase, with protein synthesis being similar to control values as well as the mass of the colon. This was closely associated with the improvement in colon integrity and the protective effect of CNRZ160 on the inflammatory colonic condition. As a result, the AA sequestration by the gut could be reduced and the nutritional AA bioavailability better maintained for the muscle in order to optimize muscle protein accretion. As the capacity of muscle and splanchnic area to exchange nutrients depending on gut/liver AA requirements remains efficient during aging (38), an increased supply of AA from muscle to sustain gut protein synthesis was not required anymore and may then explain the decreased proteolysis we recorded in skeletal muscle with CNRZ160 supplementation.

## Conclusion

We have clearly shown that a dietary strategy with probiotics can be considered as an efficient way to prevent muscle mass loss occurring during low-grade inflammation in a context of aging. Our data consolidate the concept of the existence of a gut-muscle cross-talk (18, 39) and pave the way to probiotic strategies that could limit sarcopenia development by targeting both gut and systemic inflammation. Such strategies could be even more valuable in the human frail population that can be reluctant to current recommendations of physical exercise or protein-energy supplements. By limiting inflammation, CNRZ160 can act as an optimizer of the effect of dietary proteins-whose supply is sometimes insufficient in old population - that are known to stimulate muscle anabolism. This anti-inflammatory probiotic strategy should then be considered as an ancillary strategy capable to enhance efficiently the dietary effect of proteins on muscle in low-grade inflamed old population. Consequently, on a long-term basis, the combination of already existing strategies (protein or AA supplements) with probiotic supplementation as CNRZ160 could be considered, and particularly *via* the production of fermented dairy products that contain both proteins and probiotic bacteria. As *S. thermophilus* is already present in yogurt, the optimization of such products is indeed a possible option.

## Data availability statement

The original contributions presented in the study are included in the article/supplementary material, further inquiries can be directed to the corresponding author.

## Ethics statement

The animal study was reviewed and approved by Comité Ethique Auvergne.

## Author contributions

IS-A and DD were responsible for study design, data collection, data analysis, and drafting of the manuscript. J-MC was responsible for study design and drafting of the manuscript. CM, J-LK, L-NG, MA, and VM were responsible for data collection. FM and PL were involved in manuscript drafting. All authors contributed to the article and approved the submitted version.

## Funding

The study was financed by Institut Carnot Qualiment, Agence Nationale pour la Recherche and INRAE.

## Conflict of interest

The authors declare that the research was conducted in the absence of any commercial or financial relationships that could be construed as a potential conflict of interest.

## Publisher's note

All claims expressed in this article are solely those of the authors and do not necessarily represent those of their affiliated organizations, or those of the publisher, the editors and the reviewers. Any product that may be evaluated in this article, or claim that may be made by its manufacturer, is not guaranteed or endorsed by the publisher.

## References

[1] Gibson JA Crowe S. Frailty in critical care: examining implications for clinical practices. Crit Care Nurse. (2018) 38:29–35. 10.4037/ccn2018336 29858193

[2] Dent E Morley JE Cruz-Jentoft AJ Woodhouse L Rodríguez-Mañas L Fried LP. Physical frailty: ICFSR international clinical practice guidelines for identification and management. J Nutr Health Aging. (2019) 23:771–87. 10.1007/s12603-019-1273-z 31641726 PMC6800406

[3] Cruz-Jentoft AJ Dawson Hughes B Scott D Sanders KM Rizzoli R. Nutritional strategies for maintaining muscle mass and strength from middle age to later life: a narrative review. Maturitas. (2020) 132:57–64. 10.1016/j.maturitas.2019.11.007 31883664

[4] Tournadre A Vial G Capel F Soubrier M Boirie Y. Sarcopenia. Joint Bone Spine. (2019) 86:309–14. 10.1016/j.jbspin.2018.08.001 30098424

[5] Franceschi C Garagnani P Parini P Giuliani C Santoro A. Inflammaging: a new immune-metabolic viewpoint for age-related diseases. Nat Rev Endocrinol. (2018) 14:576–90. 10.1038/s41574-018-0059-4 30046148

[6] Argilés JM Busquets S Stemmler B López-Soriano FJ. Cachexia and sarcopenia: mechanisms and potential targets for intervention. Curr Opin Pharmacol. (2015) 22:100–6. 10.1016/j.coph.2015.04.003 25974750

[7] Michaud M Balardy L Moulis G Gaudin C Peyrot C Vellas B. Proinflammatory cytokines, aging, and age-related diseases. J Am Med Dir Assoc. (2013) 14:877–82. 10.1016/j.jamda.2013.05.009 23792036

[8] Buffiere C Mariotti F Savary-Auzeloux I Migne C Meunier N Hercberg S. Slight chronic elevation of C-reactive protein is associated with lower aerobic fitness but does not impair meal-induced stimulation of muscle protein metabolism in healthy old men. J Physiol. (2015) 593:1259–72. 10.1113/jphysiol.2014.286054 25557160 PMC4358683

[9] Visser M Pahor M Taaffe DR Goodpaster BH Simonsick EM Newman AB. Relationship of interleukin-6 and tumor necrosis factor-alpha with muscle mass and muscle strength in elderly men and women: the Health ABC Study. J Gerontol A Biol Sci Med Sci. (2002) 57:M326–332. 10.1093/gerona/57.5.M326 11983728

[10] Rieu I Magne H Savary-Auzeloux I Averous J Bos C Peyron MA. Reduction of low grade inflammation restores blunting of postprandial muscle anabolism and limits sarcopenia in old rats. J Physiol. (2009) 587:5483–92. 10.1113/jphysiol.2009.178319 19752122 PMC2793878

[11] Voisin L Breuillé D Ruot B Rallière C Rambourdin F Dalle M. Cytokine modulation by PX differently affects specific acute phase proteins during sepsis in rats. Am J Physiol. (1998) 275:R1412–9. 10.1152/ajpregu.1998.275.5.R1412 9791055

[12] Claesson MJ Jeffery IB Conde S Power SE O'connor EM Cusack S. Gut microbiota composition correlates with diet and health in the elderly. Nature. (2012) 488:178–184. 10.1038/nature11319 22797518

[13] Shintouo CM Mets T Beckwee D Bautmans I Ghogomu SM Souopgui J. Is inflammageing influenced by the microbiota in the aged gut? A systematic review. Exp Gerontol. (2020) 141:111079. 10.1016/j.exger.2020.111079 32882334

[14] O'toole PW Jeffery IB. Microbiome-health interactions in older people. Cell Mol Life Sci. (2018) 75:119–28. 10.1007/s00018-017-2673-z 28986601 PMC11105677

[15] Calder PC Bosco N Bourdet-Sicard R Capuron L Delzenne N Dore J. Health relevance of the modification of low grade inflammation in ageing (inflammageing) and the role of nutrition. Ageing Res Rev. (2017) 40:95–119. 10.1016/j.arr.2017.09.001 28899766

[16] Picca A Fanelli F Calvani R Mule G Pesce V Sisto A. Gut dysbiosis and muscle aging: searching for novel targets against sarcopenia. Mediators Inflamm. (2018) 2018:7026198. 10.1155/2018/7026198 29686533 PMC5893006

[17] Fransen F Van Beek AA Borghuis T Aidy SE Hugenholtz F Van Der Gaast-De Jongh C. Aged gut microbiota contributes to systemical inflammaging after transfer to germ-free mice. Front Immunol. (2017) 8:1385. 10.3389/fimmu.2017.01385 29163474 PMC5674680

[18] Ticinesi A Lauretani F Milani C Nouvenne A Tana C Del Rio D. Aging gut microbiota at the cross-road between nutrition, physical frailty, and sarcopenia: is there a gut-muscle axis? Nutrients. (2017) 9: 1303. 10.3390/nu9121303 29189738 PMC5748753

[19] Prokopidis K Chambers E Lochlainn NI Witard M. Mechanisms linking the gut-muscle axis with muscle protein metabolism and anabolic resistance: implications for older adults at risk of sarcopenia. Front Physiol. (2021) 12:770455. 10.3389/fphys.2021.770455 34764887 PMC8576575

[20] Dardevet D Mosoni L Savary-Auzeloux I Peyron MA Polakof S Rémond D. Important determinants to take into account to optimize protein nutrition in the elderly: solutions to a complex equation. Proc Nutr Soc. (2021) 80:207–220. 10.1017/S0029665120007934 33198824

[21] Junjua M Kechaou N Chain F Awussi AA Roussel Y Perrin C. A large scale *in vitro* screening of Streptococcus thermophilus strains revealed strains with a high anti-inflammatory potential. Lwt-Food Sci Technol. (2016) 70:78–87. 10.1016/j.lwt.2016.02.006

[22] Mercier S Breuille D Mosoni L Obled C Patureau Mirand P. Chronic inflammation alters protein metabolism in several organs of adult rats. J Nutr. (2002) 132:1921–8. 10.1093/jn/132.7.1921 12097671

[23] Zou X Ji J Wang J Qu H Shu DM Guo FY. Dextran sulphate sodium (DSS) causes intestinal histopathology and inflammatory changes consistent with increased gut leakiness in chickens. Br Poult Sci. (2018) 59:166–72. 10.1080/00071668.2017.1418498 29262695

[24] Metzger CE Narayanan SA Elizondo JP Carter AM Zawieja DC Hogan HA. DSS-induced colitis produces inflammation-induced bone loss while irisin treatment mitigates the inflammatory state in both gut and bone. Sci Rep. (2019) 9:15144. 10.1038/s41598-019-51550-w 31641205 PMC6805923

[25] Chassaing B Srinivasan G Delgado MA Young AN Gewirtz AT Vijay-Kumar M. Fecal lipocalin 2, a sensitive and broadly dynamic non-invasive biomarker for intestinal inflammation. PLoS ONE. (2012) 7:e44328. 10.1371/journal.pone.0044328 22957064 PMC3434182

[26] Chatel JM Dardevet D Savary-Auzeloux I Jarzaguet M David J Dary-Mourot A. Souche de *Streptococcus termophilus* CNRZ160 pour le traitement et la prévention de l'inflammation intestinale et des désordres associés, chez un individu. International patent WO/2020/020540 (2020).

[27] Eichele DD Kharbanda KK. Dextran sodium sulfate colitis murine model: AN indispensable tool for advancing our understanding of inflammatory bowel diseases pathogenesis. World J Gastroenterol. (2017) 23:6016–29. 10.3748/wjg.v23.i33.6016 28970718 PMC5597494

[28] Dardevet D Remond D Peyron MA Papet I Savary-Auzeloux I Mosoni L. Muscle wasting and resistance of muscle anabolism: the “anabolic threshold concept” for adapted nutritional strategies during sarcopenia. Sci World J. (2012) 2012:269531. 10.1100/2012/269531 23326214 PMC3541599

[29] Boirie Y Gachon P Beaufrere B. Splanchnic and whole-body leucine kinetics in young and elderly men. Am J Clin Nutr. (1997) 65:489–95. 10.1093/ajcn/65.2.489 9022534

[30] Powell-Tuck J. Protein metabolism in inflammatory bowel disease. Gut. (1986) 27(Suppl 1):67–71. 10.1136/gut.27.Suppl_1.67 3539713 PMC1434623

[31] Mosoni L Houlier ML Mirand PP Bayle G Grizard J. Effect of amino-acids alone or with insulin on muscle and liver protein-synthesis in adult and old rats. Am J Physiol. (1993) 264:E614–20. 10.1152/ajpendo.1993.264.4.E614 8476040

[32] Balage M Averous J Remond D Bos C Pujos-Guillot E Papet I. Presence of low-grade inflammation impaired postprandial stimulation of muscle protein synthesis in old rats. J Nutr Biochem. (2010) 21:325–31. 10.1016/j.jnutbio.2009.01.005 19369058

[33] Lang CH Frost RA Vary TC. Regulation of muscle protein synthesis during sepsis and inflammation. Am J Physiol Endocrinol Metab. (2007) 293:E453–459. 10.1152/ajpendo.00204.2007 17505052

[34] Sartori R Romanello V Sandri M. Mechanisms of muscle atrophy and hypertrophy: implications in health and disease. Nat Commun. (2021) 12:330–330. 10.1038/s41467-020-20123-1 33436614 PMC7803748

[35] Peris-Moreno D Cussonneau L Combaret L Polge C Taillandier D. Ubiquitin ligases at the heart of skeletal muscle atrophy control. Molecules. (2021) 26:407. 10.3390/molecules26020407 33466753 PMC7829870

[36] Saul D Kosinsky RL. Dextran sodium sulfate-induced colitis as a model for sarcopenia in mice. Inflamm Bowel Dis. (2020) 26:56–65. 10.1093/ibd/izz127 31228348

[37] Martín R Chain F Miquel S Motta JP Vergnolle N Sokol H. Using murine colitis models to analyze probiotics-host interactions. FEMS Microbiol Rev. (2017) 41:S49–70. 10.1093/femsre/fux035 28830096

[38] Moreau K Walrand S Boirie Y. Protein redistribution from skeletal muscle to splanchnic tissue on fasting and refeeding in young and older healthy individuals. J Am Med Dir Assoc. (2013) 14:696–704. 10.1016/j.jamda.2013.06.005 24011660

[39] Watson MD Cross BL Grosicki GJ. Evidence for the contribution of gut microbiota to age-related anabolic resistance. Nutrients. (2021) 13:706. 10.3390/nu13020706 33672207 PMC7926629

